# Intestinal macrophages arising from CCR2^+^ monocytes control pathogen infection by activating innate lymphoid cells

**DOI:** 10.1038/ncomms9010

**Published:** 2015-08-13

**Authors:** Sang-Uk Seo, Peter Kuffa, Sho Kitamoto, Hiroko Nagao-Kitamoto, Jenna Rousseau, Yun-Gi Kim, Gabriel Núñez, Nobuhiko Kamada

**Affiliations:** 1 Department of Pathology and Comprehensive Cancer Center, University of Michigan Medical School, 1500 E Medical Center Dr Ann Arbor, Michigan 48109, USA; 2 Division of Gastroenterology, Department of Internal Medicine, University of Michigan Medical School, 1150 W Medical Center Dr, Ann Arbor, Michigan 48109, USA

## Abstract

Monocytes play a crucial role in antimicrobial host defence, but the mechanisms by which they protect the host during intestinal infection remains poorly understood. Here we show that depletion of CCR2^+^ monocytes results in impaired clearance of the intestinal pathogen *Citrobacter rodentium*. After infection, the *de novo* recruited CCR2^+^ monocytes give rise to CD11c^+^CD11b^+^F4/80^+^CD103^−^ intestinal macrophages (MPs) within the lamina propria. Unlike resident intestinal MPs, *de novo* differentiated MPs are phenotypically pro-inflammatory and produce robust amounts of IL-1β (interleukin-1β) through the non-canonical caspase-11 inflammasome. Intestinal MPs from infected mice elicit the activation of RORγt^+^ group 3 innate lymphoid cells (ILC3) in an IL-1β-dependent manner. Deletion of IL-1β in blood monocytes blunts the production of IL-22 by ILC3 and increases the susceptibility to infection. Collectively, these studies highlight a critical role of *de novo* differentiated monocyte-derived intestinal MPs in ILC3-mediated host defence against intestinal infection.

## Abstract (web summary)

Monocytes are important for antimicrobial host defence in the intestine but the mechanism behind their protective function is not fully understood. Seo *et al.* show that intestinal macrophages derived from CCR2^+^ monocytes support clearance of pathogenic *Citrobacter rodentium* through activation of group 3 innate lymphoid cells.

## Graphical abstract

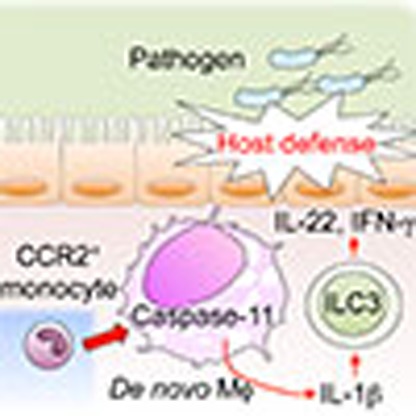

Intestinal resident mononuclear phagocytes, including macrophages (MPs) or dendritic cells (DCs), are innate immune cells that play a central role in gastrointestinal homoeostasis^1^. Intestinal MPs are defined as cells that express major histocompatibility complex (MHC) class II, CD11b, the pan-MP marker F4/80, and a receptor for fractalkine CX_3_CR1 while lacking α_E_ integrin CD103 (refs 2, 3). Intestinal MPs display immune-regulatory properties that are critical for host tolerance to innocuous antigens, such as dietary antigens as well as commensal microorganisms. For example, IL-10 robustly produced by intestinal MPs contributes to dampening of mucosal inflammation as well as the differentiation and maintenance of regulatory T cells in the intestine^2^,^3^,^4^. Although most tissue resident MPs are believed to arise from progenitors during embryonic development and are generated by self-renewal in resident tissues, the maintenance of intestinal MPs requires constant *de novo* migration of Ly6C^hi^ CCR2^+^ blood monocytes to intestinal tissues for replenishment^1^,^3^,^5^,^6^. In the presence of enteric infection, blood Ly6C^hi^ CCR2^+^ monocytes are recruited to the sites of infection where they promote inflammatory responses that facilitate pathogen clearance^7^,^8^,^9^. How recruited monocytes phenotypically switch from ‘immune-regulatory’ to ‘immune-stimulatory’ in the intestine following infection, and the significance of such a timely phenotypic change, however, remain poorly understood.

Recent evidence suggests that a subset of innate-type lymphocytes, known as innate lymphoid cells (ILCs), play a crucial role in protecting the host from intestinal infections^10^,^11^. These ILCs differ from B and T cells, in that they lack antigen receptors. However, because ILCs share developmental and functional similarities with T cells, ILCs have been categorized into three major groups based on their cytokine profile and the transcription factors required for their development: T-bet^+^ group 1 ILCs (ILC1) akin to T helper (Th1) cells that produce interferon-γ (IFNγ) and tumour-necrosis factor-α (TNFα), GATA-3^+^ group 2 ILCs (ILC2) are similar to Th2 cells and produce IL-5, IL-9 and IL-13, and RORγt^+^ group 3 ILCs (ILC3) that produce IL-17 and/or IL-22 (refs 12, 13, 14, 15). IL-22 produced by ILC3 plays a key role in ILC-mediated host defence against intestinal infection by enhancing intestinal epithelial barrier function^11^,^16^. Although the mechanisms leading to ILC activation during intestinal infection remain poorly understood, intestinal MPs and/or DCs are capable of inducing the production of IL-22 by ILC3 through IL-23 and IL-1β (refs 17, 18, 19, 20). However, it remains largely unknown how these tissue resident MPs and/or DCs acquire the ability to activate ILCs, since resident intestinal MPs are hyporesponsive to microbial stimulation and poorly produce IL-23 and IL-1β (refs 3, 4).

In this study, we provide evidence that CCR2^+^ monocytes are recruited to the intestinal lamina propria (LP) upon pathogen infection and rapidly give rise to a subset of intestinal MPs *in situ* that activate RORγt^+^ ILC3s through caspase-11 inflammasome-dependent IL-1β production. Our findings demonstrate how microbial signals from the pathogen shape the differentiation of newly recruited monocytes into ‘inflammatory’ intestinal MPs that ultimately licenses them to clear the pathogen through activation of RORγt^+^ ILC3s. Thus, our study highlights a concerted effort by two critical innate immune cell types to orchestrate host defence against pathogen infection in the intestine.

## Results

### Monocyte recruitment is critical for ILCs activation

**Figure 1:**
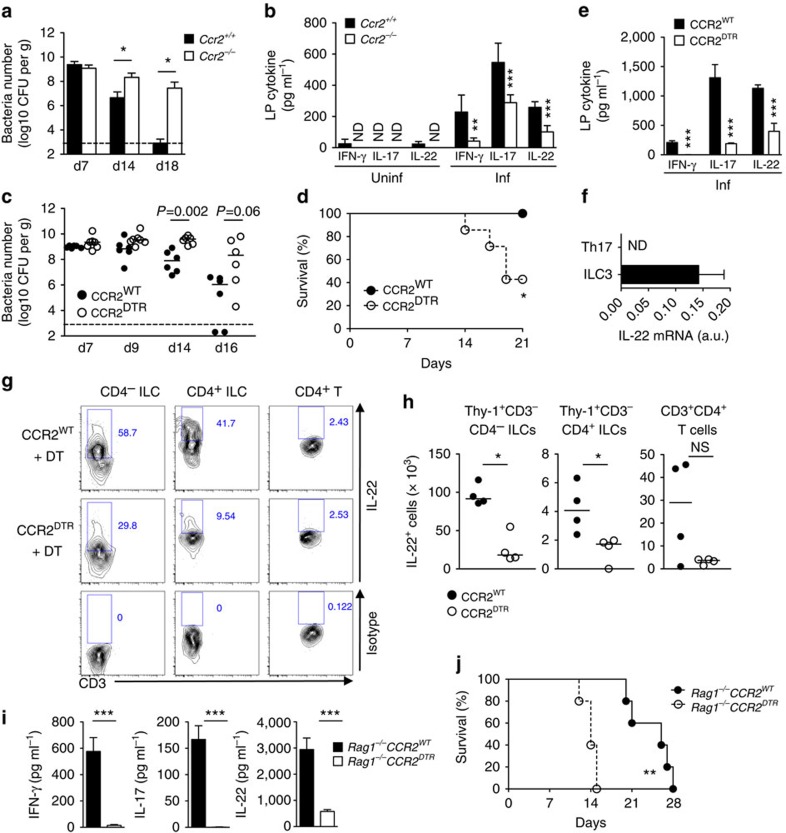
Recruitment of CCR2^+^ monocytes activate ILCs during intestinal infection. (**a**) *C. rodentium* burden in faeces from *Ccr2*^+/+^ and *Ccr2*^*−/−*^ mice. Data represent mean±s.d. (*n*=5, representative of two independent experiments). (**b**) Colonic lamina propria mononuclear cells (LPMCs) were isolated from at 0 and 12 days post infection (dpi), and cultured for 24 h. Produced cytokines were measured by ELISA. Data represent mean±s.d. (*n*=5 from two individual experiments). ND, not detected. (**c,d**) CCR2^WT^ and CCR2^DTR^ mice were infected with *C. rodentium* and diphtheria toxin (DT; 10 ng g^−1^ body weight) was injected on 5, 7, 9, 11 dpi. Bacterial burden (**c**) and mouse mortality (**d**) are shown. Dots represent individual mice. Results are representative of two independent experiments. (**e**) Cytokines produced by LPMC at 8 dpi. Data represent mean±s.d. (*n*=3, representative of three independent experiments). (**f**) RORγt^GFP/+^ mice were infected with *C. rodentium*. LPMCs were isolated on 8 dpi and Th17 (CD3^+^CD4^+^RORγt^+^) and ILC3 (CD3^−^RORγt^+^) were purified by sorting. IL-22 mRNA expression was assessed by qPCR. Data represent mean±s.d. (*n*=3, representative of 2 independent experiments). (**g,h**) CCR2^WT^ and CCR2^DTR^ mice were infected with *C. rodentium* and DT was injected on days 5 and 7. LPMCs were isolated on 8 dpi, and cultured in the presence of heat-killed *C. rodentium* (MOI=10) for 16 h. IL-22 production in CD4^−^ ILCs (Lin^-^Thy-1^+^CD3^-^CD4^−^), CD4^+^ ILCs (Lin^-^Thy-1^+^CD3^−^CD4^+^) and CD4^+^ T cells (Thy-1^+^CD3^+^CD4^+^) was assessed by flow cytometry (**g**), and absolute number of IL-22-producing T cells and ILCs are shown in **h**. (**i**) *Rag1*^*−/−*^CCR2^WT^ and *Rag1*^*−/−*^CCR2^DTR^ mice were infected with *C. rodentium* and DT was injected on days 5 and 7. LPMCs were isolated on 8 dpi and cultured for 24 h. Cytokines were measured by ELISA. Data represent mean±s.d. (*n*=4, representative of two independent experiments). (**j**) Mouse mortality of *Rag1*^*−/−*^CCR2^WT^ and *Rag1*^*−/−*^CCR2^DTR^ mice (*n*=5) infected with *C. rodentium*. DT was injected on day 5, 7, 9 and 11 post infection. NS, not significant, *P<0.05, **P<0.01, ***P<0.001 by Mann–Whitney *U*-test (**a**–**c**), Student’s *t*-test (**e,h,i**) and Log-rank test (**d,j**).

To assess the role of recruited monocytes in the control of intestinal infection, *Citrobacter rodentium* was orally administered to either wild-type (WT) mice and mice deficient in CCR2, an essential chemokine receptor for emigration of Ly6C^hi^ monocytes from the bone marrow (BM) and recruitment to the intestine^21^. Compared with WT mice, the clearance of *C. rodentium* was markedly impaired in *Ccr2*^*−/−*^ mice on day 14 and 18 (Fig. 1a), in agreement with previous results^9^. The production of IFN-γ, IL-17A and IL-22 by colonic LP cells was reduced in *Ccr2*^*−/−*^ mice on day 12 post infection (Fig. 1b). Consistent with impaired IL-22 production, expression of RegIIIβ and RegIIIγ, two known downstream targets of IL-22 (refs 11, 16), in intestinal epithelial cells was significantly decreased in *Ccr2*^*−/−*^ mice (Supplementary Fig. 1). Together, these results suggested that the recruitment of monocytes to the intestine regulates the activation of lymphocyte responses in response to pathogen infection. To exclude the possibility that developmental defects contributed to the phenotype in *Ccr2*^*−/−*^ mice, we used conditional CCR2-diphtheria toxin receptor (DTR)-depleter mice in which CCR2^+^ monocytes and monocyte-derived CCR2^+^ cells are selectively deleted after diphtheria toxin (DT) administration^22^. To assess the role of CCR2^+^ monocytes in *C. rodentium* infection, CCR2^DTR/+^ and WT littermates (CCR2^WT^) mice were infected with the pathogen followed by administration of DT. CCR2^WT^ mice, where monocytes were unaffected after DT treatment, cleared the pathogen from the intestine, and none of the animals succumbed to infection (Fig. 1c,d). In contrast, CCR2^DTR^ mice where monocytes were depleted displayed impaired eradication of *C. rodentium* on days 14 and 16, and ∼60% succumbed by day 21 post infection (Fig. 1c,d). As observed in *Ccr2*^*−/−*^ mice, mucosal IFN-γ, IL-17A and IL-22 production was significantly reduced in CCR2^DTR^ mice when compared with control CCR2^WT^ littermates (Fig. 1e). IL-22 is produced by both T cells and ILCs, and plays a critical role in protecting the host against *C. rodentium*^11^,^16^. To identify the cellular source of IL-22 during *C. rodentium* infection, Th17 cells (RORγt^+^CD3^+^CD4^+^) and ILC3s (RORγt^+^CD3^−^) were sorted from the intestines of *C. rodentium*-infected mice, and IL-22 expression in these lymphoid subsets was assessed by intracellular staining. ILC3 produced greater levels of IL-22 than Th17 cells (Fig. 1f). To determine the role of monocyte recruitment in the production of IL-22 by ILC3 cells during *C. rodentium* infection, CCR2^WT^ and CCR2^DTR^ mice were injected with DT after *C. rodentium* infection. Consistent with the results in Fig. 1f, mucosal ILCs (Lin^−^Thy-1^+^CD3^-^CD4^−^ and Lin^−^Thy-1^+^CD3^−^CD4^+^) produced IL-22 on day 8 post infection (Fig. 1g). Notably, the production of IL-22 was blunted in CCR2^DTR^ mice compared with control CCR2^WT^ littermates (Fig. 1g). Likewise, the absolute number of IL-22-producing ILCs was significantly decreased in CCR2^DTR^ mice, while IL-22-producing T cells exhibited a trend, but not a significant reduction in CCR2^DTR^ mice (Fig. 1h). To further assess the role of monocytes in the activation of ILCs, we generated CCR2^DTR^ mice in a *Rag1*^*−/−*^ background in which the main source of IL-22 are expected to be CD3^−^ ILCs. Consistently, LP cells from *Rag1*^*−/−*^CCR2^WT^ mice produced robust amounts of IL-22 after *C. rodentium* infection, suggesting that ILCs are the major source of mucosal IL-22 in response to pathogen infection (Fig. 1i). The production of IL-22, IFNγ and IL-17A were blunted in *Rag1*^*−/−*^CCR2^DTR^ mice injected with DT compared with *Rag1*^*−/−*^CCR2^WT^ mice, indicating that monocytes are also critical for production of these cytokines in the absence of T cells (Fig. 1i). To determine the role of monocytes for host defence in the absence of T cells, we infected *Rag1*^*−/−*^CCR2^DTR^ and *Rag1*^*−/−*^ mice, and monitored mouse survival over time. The mortality of *Rag1*^*−/−*^CCR2^DTR^ pretreated with DT was accelerated compared with *Rag1*^*−/−*^CCR2^WT^ mice (Fig. 1j). Collectively, these results indicate that monocytes recruited to the intestine are critical for the activation of mucosal ILC3 and host defence during *C. rodentium* infection.

### Recruited monocytes give rise to intestinal macrophages

**Figure 2:**
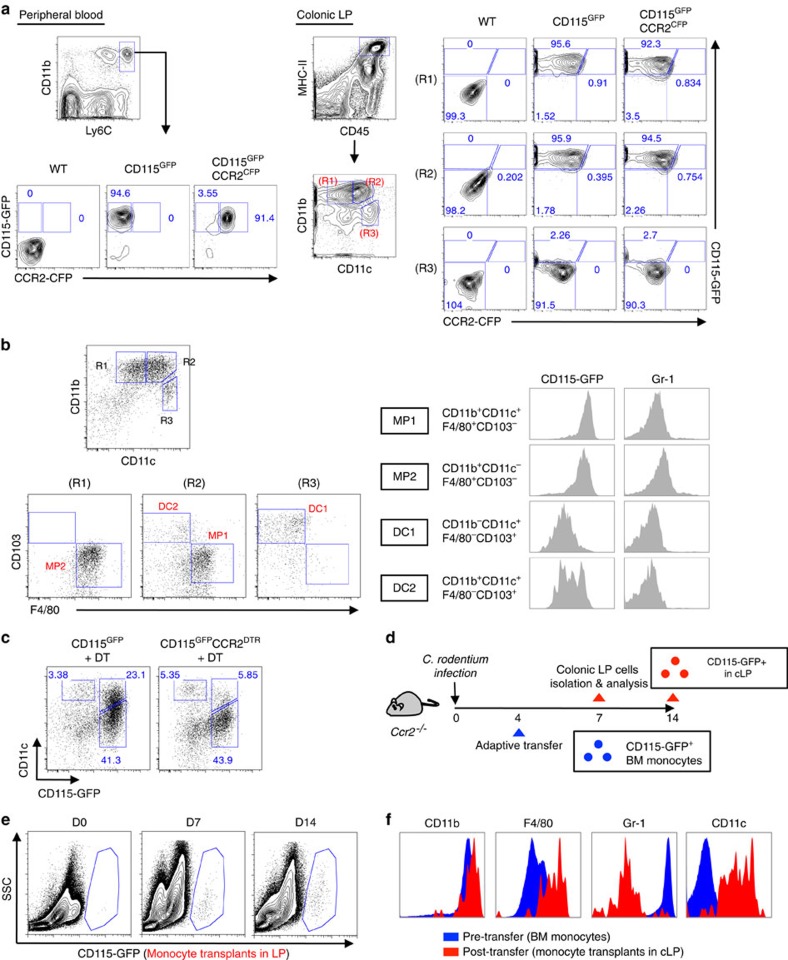
Recruited monocytes give rise to intestinal CD115^hi^CD11c^hi^ CCR2^−^ macrophages during infection. (**a**) Peripheral blood and colonic LP cells were isolated from CD115^GFP^ and CD115^GFP^CCR2^CFP^ mice. Analysis of CD45^+^CD11b^+^Ly6C^hi^ monocytes in peripheral blood and CD45^+^MHC-II^+^CD115^GFP^ cells in colonic LP. (**b**) CD45^+^MHC-II^+^ colonic mononuclear phagocytes from CD115^GFP^ mice were further analysed by flow cytometry. (**c**) CD115^GFP^ and CD115^GFP^CCR2^CFP-DTR^ mice were infected with *C. rodentium* and DT (10 ng g^−1^ body weight) was injected on days 5 and 7. LPMC were isolated on day 8 post infection and CD45^+^MHC-II^+^Gr-1^−^ colonic mononuclear phagocytes were analysed. (**d**) *Ccr2*^*−/−*^ mice were infected with *C. rodentium*. On day 4 post infection, CD11b^+^Ly6C^hi^ BM monocytes were isolated from CD115^GFP^ mice and transferred into *Ccr2*^*−/−*^ recipient mice. The presence of GFP^+^ cells in the colonic LP on days 7 and 14 post *C. rodentium* infection (days 3 and 10 post monocyte transfer) was assessed (**e**). (**f**) The surface marker profile of purified BM monocytes (preinfection) and monocytes recovered from the colonic LP on day 7 post *C. rodentium* infection (day 3 post monocyte transfer). Results are representative of at least two independent experiments.

CCR2^+^ monocytes in peripheral blood express Ly6C and CSF-1R (also known as the macrophage colony-stimulating factor (M-CSF) receptor or CD115)^23^,^24^. Although certain subsets of LP mononuclear phagocytes arise from CCR2^+^Ly6C^hi^ blood monocytes, these monocyte-derived LP mononuclear phagocytes do not express CCR2 in the intestine^23^,^24^. Consistent with previous reports^25^, Ly6C^hi^ monocytes in peripheral blood expressed both CCR2 and CD115 (Fig. 2a). In contrast, CD115^+^ cells in the intestinal LP did not express CCR2 (Fig. 2a), suggesting that CCR2 is downregulated in the intestinal LP. We therefore used CD115 as a marker of monocytes and monocyte-derived mononuclear phagocytes in the intestine instead of CCR2 and used CD115-GFP reporter mice to analyse CCR2^+^ monocyte-derived mononuclear phagocytes subsets^26^. To characterize CD115^+^ and CD115^−^ mononuclear phagocyte subsets in the intestinal LP, total mononuclear cells were isolated from the intestinal LP and CD45^+^MHC-II^+^ mononuclear phagocytes were further classified into four subsets based on the expression of CD11b, CD11c, F4/80 and CD103 (Fig. 2b). Consistent with previous results, two subsets of monocyte-derived mononuclear phagocytes (CD11b^+^CD11c^+^F4/80^+^CD103^−^, referred to as MP subset 1 (MP1) and CD11b^+^CD11c^-^F4/80^+^CD103^−^, referred to as MP subset 2 (MP2)) expressed the monocyte-derived cell marker CD115 (Fig. 2b). In contrast, the DC subsets, which are thought to arise exclusively from a common DC progenitor, DC1 (CD11b^−^CD11c^+^F4/80^−^CD103^+^) and DC2 (CD11b^+^CD11c^+^F4/80^−^CD103^+^) lacked CD115 expression (Fig. 2b). Both MP and DC subsets in the colon lacked Gr-1 expression, suggesting that recruited monocytes downregulate the expression of Gr-1 after they reach mucosal sites (Fig. 2b). The DC2 subset (CD11c^hi^CD11b^+^CD103^+^F4/80^−^) is relatively rare in the colon, while it is more abundant in the small intestine (Supplementary Fig. 2). On the basis of the expression of the monocyte marker CD115, the MP1 and MP2 subsets appear to arise from recruited monocytes. To confirm this, we depleted CCR2^+^ monocytes from CD115-reporter/CCR2-depleter (CD115^GFP^CCR2^DTR-CFP^) mice using DT. As expected, monocyte depletion led to a reduction of the MP1 subset (Supplementary Fig. 3). We then confirmed this result in *C. rodentium*-infected mice. Consistent with the results in uninfected mice, monocyte depletion affected the CD115^+^CD11c^+^ MP1 subset but did not impact on the number of MP2 and DC1 cells, suggesting that recruited monocytes preferentially give rise to the MP1 subset during infection (Fig. 2c). To further verify this, CD115-GFP^+^ monocytes were purified from the BM and transferred into *C. rodentium*-infected *Ccr2*^*−/−*^ recipient mice on day 4 post infection (Fig. 2d). The transferred CD115-GFP^+^ monocyte-derived cells were found in the colonic LP on days 3 and 10 after monocyte transfer (days 7 and 14 post infection), but not in the spleen or mesenteric lymph nodes (Fig. 2e and Supplementary Fig. 4). These results indicate that monocytes preferentially migrate to the colonic LP in infected mice. Notably, while isolated monocytes expressed CD11b and Gr-1, and lacked CD11c before the transfer, the CD115-GFP^+^ monocytes recovered from the colonic LP expressed CD11c but downregulated Gr-1 expression (Fig. 2f), indicating that monocytes differentiate into the MP1 subset after migration to the colonic LP.

### The MP1 subset is a major producer of IL-1β and IL-23

**Figure 3:**
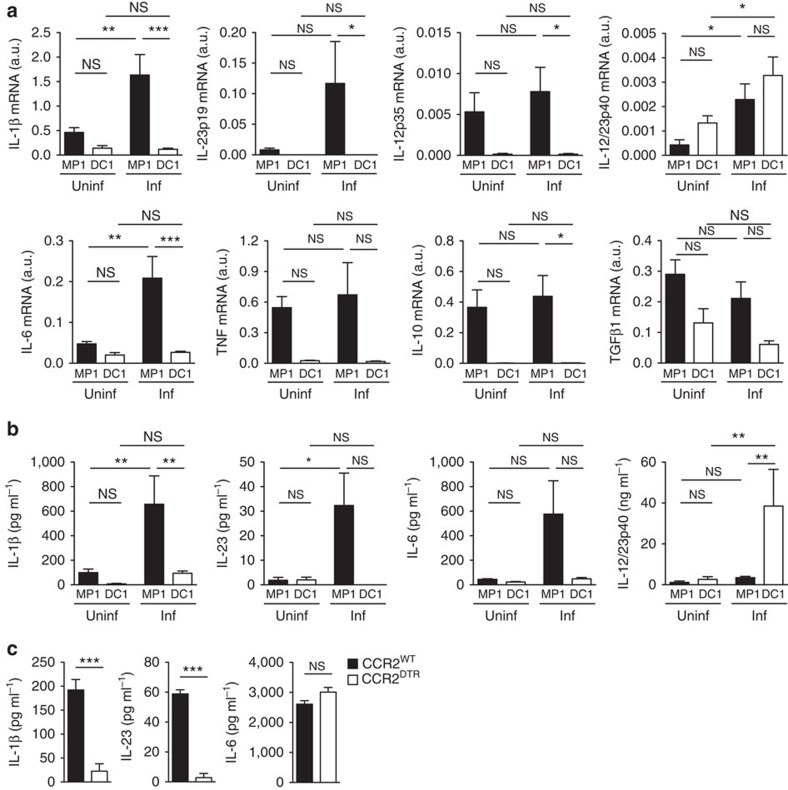
Monocyte-derived intestinal macrophages are major producers of IL-1β and IL-23. (**a**) MP1 and DC1 subsets were isolated from uninfected (uninf) and *C. rodentium*-infected (inf) CD115^GFP^ animals. Cytokine mRNA expression was analysed by qPCR. Data are given as mean±s.d. (*n*=5–7). *P<0.05; **P<0.01; ***P<0.001; NS, not significant by Dunn’s test. (**b**) Purified MP1 and DC1 subsets (1 × 10^6^ cells per ml) were cultured for 24 h without stimulation. Cytokines in the culture supernatant were analysed by ELISA. Data are given as mean±s.d. (*n*=4–6). (**c**) LPMCs were isolated from *C. rodentium*-infected CCR2^WT^ and CCR2^DTR^ mice. 2 × 10^6^ cells per ml LPMCs were cultured for 24 h without stimulation. Cytokines in the culture supernatant were analysed by ELISA. Data are given as mean±s.d. (*n*=3, representative of three independent experiments). *** *P*<0.001; NS, not significant by Student’s *t*-test.

Since our data demonstrated that newly recruited monocytes give rise to the MP1 subset *in situ* during infection, we next assessed how the MP1 subset regulates host defence during infection. We first compared the cytokine profile of the MP1 subset and the ‘non-monocyte-derived’ CD103^+^ DC1 subset which is known to play an important role in the induction of IL-22 in response to flagellin injection^17^. The MP1 and DC1 subsets were purified from the intestines of *C. rodentium*-infected and -uninfected mice to assess their cytokine profiles. As shown in Fig. 3a, the MP1 subset from infected mice expressed higher mRNA levels of pro-inflammatory cytokines, including IL-1β, IL-23p19 and IL-6, than MP1 cells from uninfected mice or the DC1 subset from uninfected or infected mice. Although most cytokines were found to be expressed at higher levels in MP1 compared with DC1 cells, IL-12/23p40 mRNA levels were higher in the DC1 subset (Fig. 3a). Consistently, MP1 cells from infected intestines produced higher amounts of IL-1β and IL-23 than MP1 cells from naive mice or the DC1 subset, while IL-12/23p40 production was much higher in the DC1 subset (Fig. 3b). To address whether the CCR2^+^ monocyte-derived MP1 subset is the major source of IL-1β and IL-23 during *C. rodentium* infection, CCR2^WT^ and CCR2^DTR/+^ mice were infected with *C. rodentium*, and cytokine production by colonic LP cells was evaluated. The production of IL-1β and IL-23, but not IL-6, was almost abrogated in CCR2^DTR/+^ mice treated with DT (Fig. 3c), indicating that the CCR2^+^ monocyte-derived MP1 subset is the major producer of IL-1β and IL-23 during *C. rodentium.*

### The MP1 subset activates ILC3 via IL-1β

**Figure 4:**
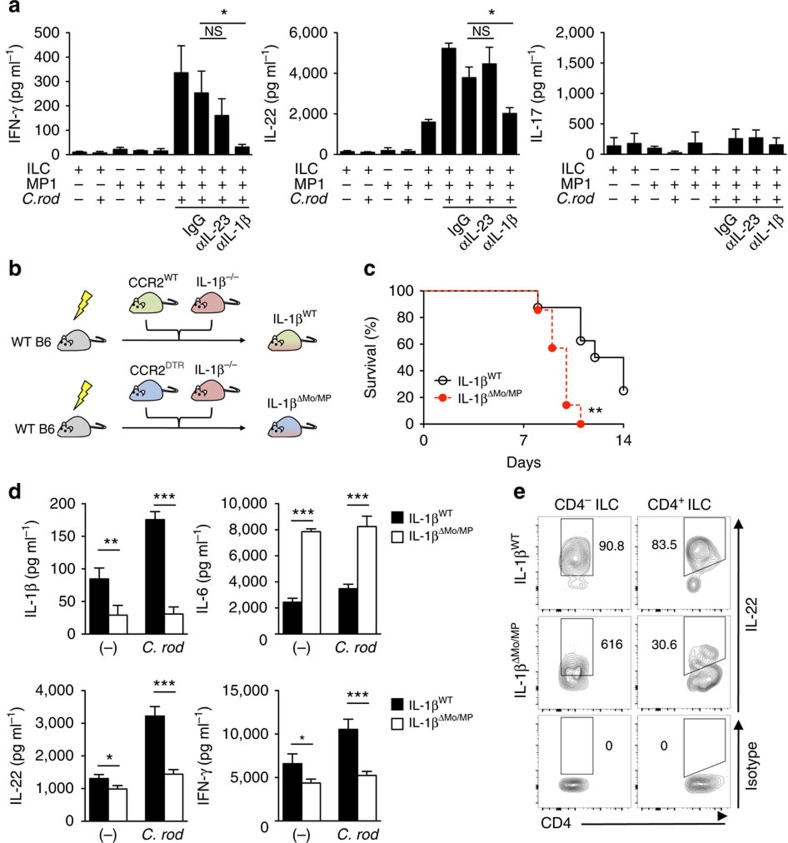
Activation of RORγt^+^ ILCs requires IL-1β produced by monocyte-derived intestinal macrophages during *C. rodentium* (*C. rod*) infection. (**a**) CD3^-^RORγt^+^ ILCs from uninfected RORγt^GFP/+^ reporter mice and MP1 cells from *C. rodentium*-infected CD115^GFP^ were isolated. ILCs and MP1 cells (1 × 10^6^ cells per ml) were cultured alone or co-cultured with or without stimulation with heat-killed *C. rodentium* (MOI=10) for 24 h. Neutralizing antibodies for IL-23 (10 μg ml^−1^), IL-1β (10 μg ml^−1^), or isotype controls were used to block cytokines. Data are given as mean±s.d. (*n*=4). *P<0.05; NS, not significant by Dunnett’s test (compared with isotype control). (**b**) Schematic illustrating experimental protocol of mixed BM chimera for IL-1β monocyte/macrophage-conditional depletion. Lethally irradiated C57BL/6 recipient mice were reconstituted with mixed bone-marrows from *Ccr2*^WT^ or *Ccr2*^DTR^ and *Il1b*^*−/−*^ mice (1:1 ratio) for 8 weeks. After 8 weeks, mice were infected with *C. rodentium*, and CCR2^+^ monocytes and monocyte-derived MP1 cells were depleted by DT injection (10 ng g^−1^ body weight) on days 5, 7, 9 and 11 post infection. (**c**) Survival of chimeric mice infected with *C. rodentium* (*n*=7). **P<0.01 by Log-rank test. Results are pooled data of two independent experiments with 3–4 mice each. (**d**) CCR2^WT^/*Il1b*^*−/−*^ control chimera (IL-1β^WT^) and CCR2^DTR^/*Il1b*^*−/−*^ chimera (IL-1β^ΔMo/MP^) were infected with *C. rodentium* and DT was injected (10 ng g^−1^ body weight) on days 5 and 7. LPMCs were isolated on day 8 post infection. 2 × 10^6^ cells per ml LPMCs were cultured with or without stimulation with heat-killed *C. rodentium* (MOI=10) for 24 h. Cytokines in the culture supernatant were analysed by ELISA. Data are given as mean±s.d. (*n*=4, representative of two independent experiments). *P<0.05; **P<0.01; ***P<0.001 by Student’s *t*-test. (**e**) Isolated LPMCs in **d** were cultured in the presence of heat-killed *C. rodentium* (MOI=10) for 16 h. IL-22 production in CD4^−^ ILCs (Lin^-^Thy-1^+^CD3^−^CD4^−^) and CD4^+^ ILCs (Lin^-^Thy-1^+^CD3^-^CD4^+^) was assessed by flow cytometry. Data are representative of four individual mice.

Given that IL-1β and IL-23 are critical activators of ILCs^20^,^27^, we hypothesized that the monocyte-derived MP1 subset activates ILCs. We first tested the role of IL-1β and IL-23 in the activation of ILC3s isolated from the intestine. RORγt^+^CD3^+^CD4^+^ Th17 cells and RORγt^+^CD3^−^ ILC3s were isolated from the intestinal LP. About half of the ILC3s expressed NKp46, a marker found specifically on ILC3 but not on Th17 cells (Supplementary Fig. 5a). Purified Th17 cells and ILC3s were then stimulated with recombinant IL-1β, IL-23 and IL-1β plus IL-23. Consistent with previous reports^20^,^27^, both IL-23 and IL-1β induced IL-22 secretion by ILC3s (Supplementary Fig. 5b). Moreover, these cytokines synergistically induced IL-22 production in ILC3s (Supplementary Fig. 5b). Additionally, co-stimulation with IL-23 and IL-1β induced ILC3s to produce robust amounts of IFN-γ, while stimulation with IL-23 or IL-1β alone did not (Supplementary Fig. 5b). In contrast, Th17 cells produced higher amounts of IL-17A than ILC3s but minimal amounts of IFN-γ and IL-22, even after dual stimulation with IL-23 and IL-1β (Supplementary Fig. 5b). To address whether the monocyte-derived intestinal MP1 cells can activate ILCs through IL-23 and IL-1β, RORγt^+^CD3^−^ ILC3 and the MP1 subset were purified from the intestine, and then co-cultured *ex vivo*. Individually cultured ILC3 and MP1 cells did not produce IL-22, even after stimulation with heat-killed *C. rodentium* (Fig. 4a). However, co-culture of ILC and MP1 induced IL-22 production, which was further enhanced in the presence of *C. rodentium* (Fig. 4a). While ILC3-MP1 co-culture did not promote IFN-γ or IL-17A production, stimulation with *C. rodentium* robustly induced IFN-γ production by ILC3s in the presence of MP1 cells (Fig. 4a). Notably, the production of both IL-22 and IFN-γ by ILC3s co-cultured with MP1 was diminished by neutralizing antibody against IL-1β, but not IL-23p19 (Fig. 4a). These results demonstrate that IL-1β produced by monocyte-derived MPs is a key inducer of IL-22 and IFN-γ production by ILC3.

Next, we evaluated the importance of IL-1β produced by monocyte-derived intestinal MPs in IL-22-mediated host defence *in vivo*. To address this, we generated IL-1β CCR2^+^ monocyte/MP conditional depleter mice using mixed BM chimeras. In these experiments, CCR2^WT^ or CCR2^DTR/+^ BM cells mixed with BM cells from *Il1b*^*−/−*^ mice at a 1:1 ratio were transplanted into lethally irradiated WT mice (Fig. 4b and Supplementary Fig. 6a). When we infected mixed chimeric mice orally with *C. rodentium*, CCR2^DTR^/*Il1b*^*−/−*^ chimeric mice (IL-1β^ΔMo/MP^) were more susceptible to *C. rodentium* and succumbed faster to infection than CCR2^WT^/*Il1b*^*−/−*^ chimeric mice (IL-1β^WT^) (Fig. 4c). Although the number of monocyte-derived intestinal MPs (MHC-II^*+*^CD11b^*+*^F4/80^*+*^) was identical in both groups after DT injection (Supplementary Fig. 7), depletion of DTR-expressing monocytes affected IL-1β production by LP cells in IL-1β^ΔMo/MP^ mice, but not in IL-1β^WT^ mice (Fig. 4d and Supplementary Fig. 6b). Consistent with compromised IL-1β production in IL-1β^ΔMo/MP^ mice, IL-22 production by total LP cells was blunted, while IL-6 production was increased in IL-1β^ΔMo/MP^ mice (Fig. 4d). More importantly, IL-22 production by ILCs was blunted in IL-1β^ΔMo/MP^ mice as it was observed in monocyte-depleted mice (Fig. 4e). Notably, control chimeric animals (IL-1β^WT^) were susceptible to *C. rodentium* whereas non-chimeric WT mice were completely protected (Figs 1d and 4c). The latter might be due to reduced levels of IL-1β in control chimeric animals (IL-1β^WT^) since about half of BM cells in control chimeric animals derives from *Il1b*^*−/−*^ mice (Supplementary Fig. 6b). Thus, IL-1β produced by monocyte-derived intestinal MPs plays a crucial role in host protection against *C. rodentium* by promoting the production of IL-22 by mucosal ILCs.

### *C. rodentium* induces IL-1β via caspase-11 activation

**Figure 5:**
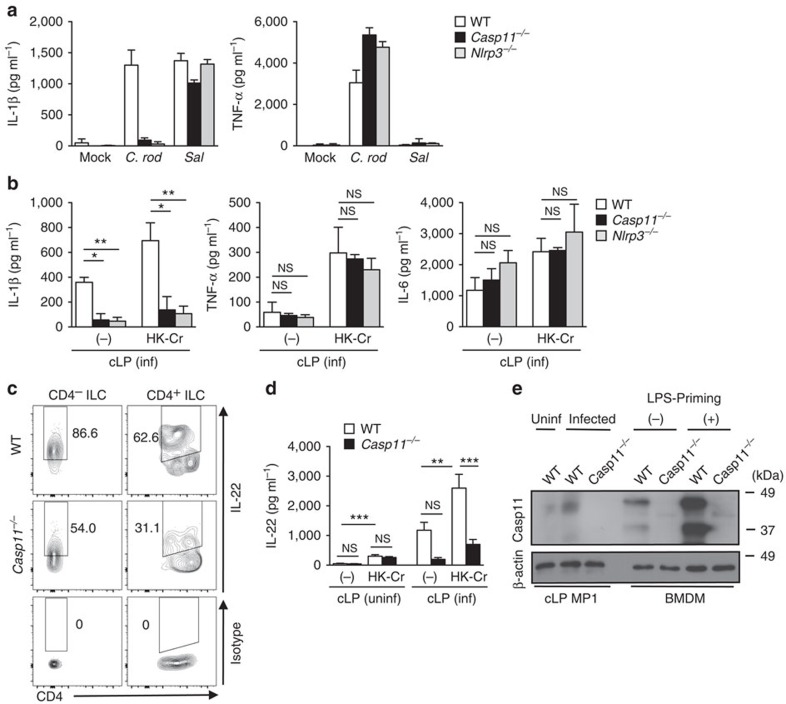
*Citrobacter rodentium* elicits IL-1β production by intestinal macrophages via caspase-11 inflammasome. (**a**) BM-derived macrophages (BMDMs) were obtained from WT, *Nlrp3*^*−/−*^, *Casp11*^*−/−*^ mice and stimulated with *C. rodentium* (*C. rod*) or *Salmonella* (*Sal*; MOI=25) for 1 h without antibiotics and then cultured additional 17 h in the presence of 100 μg ml^−1^ gentamicin. Cytokines in the culture supernatant were analysed by ELISA. Data are given as mean±s.d. (*n*=3, representative of three independent experiments). (**b**) WT, *Nlrp3*^*−/−*^ and *Casp11*^*−/−*^ mice were infected with *C. rodentium*. On day 8 post infection, LPMCs were isolated from the infected mice, and 2 × 10^6^ cells ml^−1^ LPMCs were cultured in the presence of heat-killed *C. rodentium* (MOI=10) for 24 h. Cytokines in the culture supernatant were analysed by ELISA. Data are given as mean±s.d. of 3 independent experiments. *P<0.05; **P<0.01; NS, not significant by Bonferroni test. (**c**) Isolated LPMCs in **b** were cultured in the presence of heat-killed *C. rodentium* (MOI=10) for 16 h. IL-22 production in CD4^−^ ILCs (Lin^-^Thy-1^+^CD3^-^CD4^-^) and CD4^+^ ILCs (Lin^-^Thy-1^+^CD3^-^CD4^+^) was assessed by flow cytometry. Data are representative of four individual mice. (**d**) LPMCs were isolated from uninfected and *C. rodentium*-infected (day 8 post infection) WT and *Casp11*^*−/−*^ mice and cultured in the presence of heat-killed *C. rodentium* (MOI=10) for 24 h. IL-22 in the culture supernatant was analysed by ELISA. Data are given as mean±s.e.m (*n*=4–6). **P<0.01; ***P<0.001; NS, not significant by Bonferroni test. (**e**) CD45^+^MHC-II^+^CD11b^+^CD11c^+^CD103^−^Gr-1^−^ MP1 subset was sorted from naive WT mice and *C. rodentium*-infected (day 8) WT and *Casp11*^*−/−*^ mice. 2 × 10^5^ of MP1 cells were loaded with SDS–polyacrylamide gel electrophoresis, and blotted with anti-mouse-caspase-11 antibody. As positive and negative controls, BMDMs from WT and *Casp11*^*−/−*^ mice with or without LPS priming (6 h) were used. The original gel images are shown in Supplementary Fig. 12.

Given that the intestinal MP1 subset from infected mice produces significant amounts of IL-1β, *C. rodentium* may be capable of activating an inflammasome during infection (Fig. 3). To delineate the mechanism by which monocyte-derived MP1 cells acquire the ability to produce IL-1β during *C. rodentium* infection, we first investigated the expression of inflammasome proteins in intestinal mononuclear phagocytes. The expression of *Nlrp3*, *Nlrc4*, *Casp1* and *Casp11* mRNAs were higher in MP1 than in DC1 cells, even in the steady state (Supplementary Fig. 8), suggesting that expression of inflammasome proteins may contribute to the enhanced production of IL-1β by the MP1 subset. To identify which inflammasome mediates *C. rodentium*-induced IL-1β production in MPs, BM-derived MPs (BMDMs) were stimulated with *C. rodentium*, and IL-1β production was measured in the culture supernatants. *C. rodentium*-induced IL-1β and TNF-α in BMDMs (Fig. 5a). IL-1β levels induced by *C. rodentium* were dramatically reduced in BMDMs derived from *Nlrp3*^*−/−*^ or *Casp11*^*−/−*^ mice, while TNF-α production was unaffected (Fig. 5a). In contrast, *Salmonella*-induced IL-1β, which has been shown to be dependent on the NLRC4 inflammasome^28^, was intact in *Nlrp3*^*−/−*^ or *Casp11*^*−/−*^ BMDMs (Fig. 5a). Given that caspase-11 acts upstream of the NLRP3 inflammasome^29^, these results indicated that *C. rodentium* may induce IL-1β production via caspase-11-mediated non-canonical inflammasome activation. To address the role of caspase-11 in IL-1β production *in vivo*, *Casp11*^*−/−*^ and *Nlrp3*^*−/−*^ mice were infected with *C. rodentium*, and the production of cytokines by LP cells was evaluated *ex vivo* in the absence and presence of pathogen stimulation. Production of IL-1β, TNF-α and IL-6 by LP cells was enhanced by stimulation of LP cells with heat-killed *C. rodentium* (Fig. 5b). Importantly, the production IL-1β, but not TNF-α or IL-6, by LP cells was impaired in LP cells from infected *Casp11*^*−/−*^ and *Nlrp3*^*−/−*^ mice when compared with WT mice (Fig. 5b). To address the link between caspase-11 and mucosal ILC activation, we next analysed IL-22 production by colonic ILCs during *C. rodentium* infection in *Casp11*^*−/−*^ mice. Consistent with our results, IL-22 production by ILCs was significantly impaired in *Casp11*^*−/−*^ mice (Fig. 5c). Since IL-22 induction was compromised in *C. rodentium*-infected *Casp11*^*−/−*^ mice, but not in naive *Casp11*^*−/−*^ mice (Fig. 5d), we examined the expression of caspase-11 in colonic MPs isolated from naive and *C. rodentium*-infected WT mice. Notably, the expression of caspase-11 was low or undetected in naive mice, but was clearly detected in colonic MPs of *C. rodentium*-infected mice (Fig. 5e). These results indicate that *C. rodentium* infection induces caspase-11 expression in intestinal macrophages, and caspase-11 contributes to the production of IL-1β by MPs, which promotes mucosal defence by activating ILCs.

### LEE virulence factors activate caspase-11 in macrophages

Lastly, we asked what factors are involved in caspase-11/NLRP3 activation by *C. rodentium*. BMDMs were stimulated with WT and isogenic mutant *C. rodentium* strains, and IL-1β production was assessed. Notably, deletion of *ler*, the global regulator of locus of enterocyte effacement (LEE) virulence, abrogated the ability of *C. rodentium* to induce IL-1β release, but not TNFα (Supplementary Fig. 9a). In contrast, *C. rodentium* mutants deficient in the adhesion molecule Intimin (*Δeae*), its receptor Tir (*Δtir*), and type 3 secretion system (T3SS; *ΔescN*, *ΔescU*) did not cause any defects in IL-1β production, suggesting that these Ler-regulated factors are dispensable for caspase-11 activation (Supplementary Fig. 9a). To investigate the mechanism by which Ler regulates caspase-11 activity, we determine whether *C. rodentium* releases a factor(s) that induces caspase-11 activity. As shown in Supplementary Fig. 9b, culture supernatant of WT *C. rodentium,* but not the Δ*ler* mutant strain, stimulated BMDMs to produce IL-1β in a caspase-11-dependent manner. In contrast to IL-1β, TNF-α was induced by stimulation of BMDMs with supernatant of both WT and Δ*ler* mutant (Supplementary Fig. 9b). Thus, *C. rodentium* Ler regulates the release of a factor(s) that mediates IL-1β production via caspase-11.

## Discussion

Previous studies have revealed that intestinal mononuclear phagocytes, such as CX_3_CR1-expressiong cells, regulate the production of IL-22 by ILC3 cells^18^,^19^,^20^. However, the previous works did not discriminate between resident and *de novo* recruited antigen-presenting cells. Using several approaches including CCR2-depleter (CCR2^DTR^) mice that do not have any defect in the development of intestinal MPs, we show that intestinal MPs arising *de novo* from CCR2^+^ monocytes play a pivotal role in induction of IL-22 by RORγt^+^ ILC3 cells during *C. rodentium* infection. Since the monocyte-derived MP1 subset in this study expresses CX_3_CR1 (Supplementary Fig. 10), MP1 cells appear to be identical to CX_3_CR1^+^ mononuclear phagocytes reported previously^18^,^19^,^20^. Consistently, MyD88 signalling was required for the production of IL-1β by MP1 cells (Supplementary Fig. 11) and MyD88 is important for ILC activation by CX_3_CR1^+^ MPs^20^. The *de novo* differentiated monocyte-derived CX_3_CR1^+^ MPs activate ILCs, thus playing a key role in host defence during enteric pathogen infection. In line with these studies in mice, human CD14^+^CX_3_CR1^+^ monocyte-derived intestinal MPs activate RORγt^+^ ILC3 in patients with Crohn’s disease^30^,^31^. Therefore, monocyte-derived MPs may contribute to host defence during infection as well as the pathogenesis of Crohn’s disease.

Monocyte-derived MPs are typically immune regulatory under steady-state conditions^3^,^32^. However, intestinal MPs also initiate an inflammatory response when invasive pathogens are encountered. Hence, recruited monocytes undergo differentiation that potentiates their capacity to combat infection. Although the precise signals that direct the differentiation of monocytes into MPs within the intestine have yet to be elucidated, the mucosal microenvironment in the inflamed intestine may drive the ‘inflammatory’ differentiation of MPs. For instance, recruited monocytes give rise to CD11c^+^ MPs that display a pro-inflammatory phenotype in the inflamed colon, while they primarily differentiate into a CD11c^−^ immune-regulatory subset of MPs under steady-state conditions^3^,^32^. Consistently, we found that intestinal infection causes recruited monocytes to differentiate preferentially into CD11c^+^ MPs (referred to as MP1 subset in this study) in the colonic LP. The monocyte-derived CD11c^+^ MP1 subset in the inflamed mucosa exhibited a more pro-inflammatory phenotype (for example, IL-1β and IL-23 production) than the CD11c^+^ MP1 subset residing in the normal colon, despite identical expression of surface markers (MHC-II^+^ CD11b^+^ CD11c^+^ F4/80^+^ CD103^−^ Gr-1^−^ CSF-1R^+^) associated with macrophage differentiation. This suggests that the CD11c^+^ MP1 population found in inflamed and non-inflamed mucosal tissue are phenotypically distinct. Likewise, monocyte-derived CD14^+^ MPs are identical in the intestine of Crohn’s disease patients and healthy individuals in terms of their surface markers, yet they differ in their ability to produce pro-inflammatory cytokines, with the cells isolated from the intestine of Crohn’s disease patients being more stimulatory^30^. These results suggest that the local inflammatory milieu re-programs the differentiation signals for recruited monocytes, thereby inducing their preferential differentiation into pro-inflammatory CD11c^+^ MPs rather than regulatory CD11c^−^ MPs. Although the precise ‘local cues’ remain unclear, microbial stimulation through pattern recognition receptors may play a role in the differentiation programme of recruited monocytes into inflammatory intestinal MPs. In support of this notion, we found that the MP1 subset produces inflammatory molecules including IL-1β, TNFα and IL-6 upon stimulation with *C. rodentium*. Furthermore, intestinal CD11c^−^ MPs and their Ly6C^hi^ monocyte precursors were previously shown to express TLR2 and NOD2 and produce inflammatory molecules in response to microbial ligands^32^. In addition to direct microbial stimulation, ‘inflammatory’ MP differentiation can be simultaneously influenced by local pro-inflammatory cytokines. For example, mucosal IFN-γ affects the differentiation of human intestinal MPs by promoting their pro-inflammatory activity^30^.

IL-1β plays a key role in host defence by inducing several immune events involved in pathogen eradication. For instance, IL-1β acts on both endothelial and epithelial cells to promote the recruitment of neutrophils to the site of infection^33^,^34^. Likewise, IL-1β is critical for the differentiation of Th17 and ILC3 cells in the intestine^20^,^27^,^35^. Our study shows that the monocyte-derived MP1 subset is a critical cellular source of IL-1β to promote ILC activation during *C. rodentium* infection. Although IL-1β and IL-23 synergistically enhanced the production of IL-22 by RORγt^+^ ILC3 isolated from the intestine, *in vitro* MP-ILC co-cultures and *in vivo* experiments supported that IL-1β, but not IL-23, is a key regulator of ILC function during *C. rodentium* infection. Consistent with this finding, a recent report revealed that IL-22 production by ILC3 is partly IL-23 and completely IL-1β dependent, as CX_3_CR1^+^ mononuclear phagocytes from *Il23a*^*−/−*^ mice displayed reduced but still significant activation of ILC3, while ILC3 from *Il1r*^*−/−*^ mice were greatly impaired in IL-22 production^20^. Moreover, IL-23 has been shown to be dispensable for IL-22 production by ILCs in the intestine under steady-state conditions^36^. However, previous reports suggested that IL-23 was required for IL-22 production by ILCs during infection^11^,^16^. Indeed, *Il23a*^*−/−*^ mice are more susceptible to *C. rodentium* infection^37^. Thus, further studies are needed to fully understand the role of IL-1β- and IL-23-mediated ILC3 activation in the intestine.

In addition to IL-22, IFN-γ produced by ILC3 contributes to host defence against intestinal pathogens including *C. rodentium*^38^. Indeed, our results showed that RORγt^+^ ILCs produced both IL-22 and IFN-γ *in vitro* when stimulated with IL-1β and IL-23. Notably, RORγt^+^ ILCs robustly produced IFN-γ in an IL-1β-dependent manner when they were co-cultured with MP1 cells. Thus, monocyte-derived intestinal MPs can regulate both the production of IL-22 by CCR6^+^RORγt^+^T-bet^−^ ILCs and IFN-γ by CCR6^−^RORγt^+^T-bet^+^ ILCs, and these two key cytokines may orchestrate host defence responses against infectious pathogens. Furthermore, CD14^+^CCR2^+^CX_3_CR1^+^ monocyte-derived intestinal MPs can elicit the activation of IFN-γ-producing CD3^−^CD56^+^NKp46^+^NKp44^−^ group 1 ILCs from Crohn’s disease patients^39^. Because the barrier function of the intestinal epithelium is enhanced by IL-22, but disrupted by IFN-γ (refs 40, 41, 42), balanced IFN-γ and IL-22 production by ILCs may affect the outcome of infectious and inflammatory disease, and further work is required to address how or whether intestinal MPs influence such an intricate balance. In addition to IL-22 and IFN-γ, monocyte depletion impaired the production of IL-17A by innate immune cells (Fig. 1i). Although IL-17A can be produced by isolated RORγt^+^ ILC3s (Supplementary Fig. 5b), co-culture of RORγt^+^ ILC3s with MP1 cells did not result in the production of IL-17A by ILCs (Fig. 4a). These results indicated that in *Rag1*^*−/−*^ mice, IL-17A is produced by a cell type other than ILC3s. This could be another subset of ILCs (for example, ILC1) or another type of innate immune cells (for example, neutrophils^43^,^44^,^45^). The activation of these innate immune cells might be regulated by recruited monocytes and/or monocyte-derived MPs in the intestine.

Although IL-1β is important for the regulation of ILC3 during intestinal infection^46^, the mechanism that triggers IL-1β production remains unclear. In this study, we show that intestinal MPs secrete IL-1β via the activation of the caspase-11 inflammasome in the presence of *C. rodentium*. Mounting evidence points to the importance of caspase-11 for the immune response against Gram-negative bacteria^29^,^47^. Indeed, it has been reported that the caspase-11-mediated non-canonical inflammasome activation contributes to host defence against Gram-negative enteric pathogens, such as *Salmonella* and *C. rodentium*^48^,^49^. Caspase-11 is expressed in both macrophages and epithelial cells in the gut^49^,^50^,^51^,^52^, and its expression is markedly induced by inflammation^51^. Caspase-11 in both macrophages and epithelial cells contributes to the regulation of intestinal inflammation through the production of IL-1β and IL-18 in the gut^49^,^50^,^51^,^52^. It is known that activation of the caspase-11-mediated non-canonical inflammasome in intestinal epithelial cells maintains epithelial barrier function and promotes host defence against enteric pathogens via induction of IL-18 (refs 49, 50, 51). However, it remains largely unclear how caspase-11 functions in intestinal macrophages despite the fact that a series of *in vitro* experiments suggest that caspase-11 acts in macrophages to regulate the immune response to Gram-negative bacteria^29^,^47^. In this study, we found that caspase-11 expression in intestinal MPs is low at the steady state, as it had been reported for NLRP3 (Fig. 5e)^33^, but is increased in the *de novo* differentiated MPs in the inflamed intestine (Fig. 5e). As mentioned above, inflammation regulates the expression of caspase-11 in the gut^51^. Another report suggested that reactive oxygen species, which are induced during inflammation, promotes the expression of caspase-11 in macrophages^53^. Although the precise signals that regulate caspase-11 expression in MPs remain to be better defined, our results suggest that the inflammatory milieu upregulates caspase-11 expression in *de novo* MPs which may account for their increased capacity to produce IL-1β than resident MPs. Although IL-1β production was impaired in *Casp11*^*−/−*^ mice which was linked to reduced IL-22 production, residual IL-1β and IL-22 production in the intestine was observed even in *Casp11*^*−/−*^ mice. Given the evidence that some commensal bacteria contribute to the production of IL-1β in intestinal MPs^35^,^54^, it is conceivable that commensal bacteria can contribute to IL-1β production in *Casp11*^*−/−*^ mice. Consistently, certain commensal bacteria can activate the NLRP3 inflammasome, but not the caspase-11-dependent non-canonical pathway, to produce IL-1β (ref. 55). Thus, caspase-11 in intestinal macrophages is likely to be important, particularly in early host defence against enteric pathogens. In the later stages of infection, other inflammasomes, such as NLRP3, activated by commensal bacteria, may play a more important role in the regulation of mucosal IL-1β.

Although recent findings showed the importance of intracellular lipopolysaccharides (LPS) for caspase-11 activation^29^,^47^, the mechanism by which *C. rodentium* activates caspase-11 remains unclear. Our data indicate that Ler-regulated virulence factors, but not components of the type 3 secretion system and/or adhesion molecules regulated by Ler, are involved in caspase-11 activation^56^. Notably, we found that Ler regulates the release of factor(s) by the pathogen that activates caspase-11 (Supplementary Fig. 9). Although LPS is a known activator of caspase-11 secreted by Gram-negative bacteria when it is delivered into the cytosol of macrophages, Ler may not regulate LPS secretion per se because TNF-α induction by *C. rodentium* supernatant was not impaired in the *Δler* mutant. Thus, one possible mechanism is that Ler regulates the secretion of factors(s) that contribute to delivery of LPS into the macrophage cytosol.

Collectively, the current study demonstrates the importance of newly recruited monocytes as well as the cross-talk between RORγt^+^ ILC3 and monocyte-derived intestinal MPs in host defence against intestinal infection. Since both ILC3 and intestinal MPs play vital roles in anti-inflammatory as well as pro-inflammatory responses in the intestine, understanding the complex interplay between these two types of innate immune cells may provide important insights into not only protective immunity but also the pathogenesis of inflammatory disease.

## Methods

### Mice

Wild-type C57BL/6, *Ccr2*^*−/−*^, *Nlrp3*^*−/−*^, *Casp11*^*−/−*^, *Il1b*^*−/−*^, RORγt^GFP^ reporter, CD115^GFP^ reporter mice^26^ were bred and kept under specific pathogen-free conditions in the University of Michigan Animal Facility. CCR2 reporter/depleter (CCR2^CFP/DTR^) mice were kindly provided by Dr Eric Pamer (Memorial Sloan Kettering Cancer Center, New York)^22^. Eight to 16 week old female and male mice were used for experiments. All animals were handled according to the protocols approved by the University Committee on Use and Care of Animals (UCUCA) at the University of Michigan.

### Reagents and bacteria

Ultrapure *Escherichia coli* O111:B4 LPS and CpG were from Invivogen. Recombinant mouse IL-23 and IL-1β, neutralizating antibody for IL-23p19 and isotype control (rat IgG) were purchased from eBioscience. Blocking antibody for mouse IL-1β was obtained from Leinco Technologies. Kanamycin (Km)-resistant wild-type *C. rodentium* strain DBS120 (pCRP1::Tn5) was a gift of Dr David Schauer (Massachusetts Institute of Technology, Massachusetts). The isogenic *C. rodentium* Δ*ler*, Δ*tir*, Δ*eae*, Δ*escN*, Δ*escU* mutants were generated from DBS120. *Salmonella enterica* serovar Typhimurium strain SL1344 was a gift from Dr D. Monack (Stanford University, California). For preparation of heat-killed *C. rodentium*, the pathogen was cultured overnight in Luria-Bertani (LB) medium, inoculated into DMEM at a 1:50 dilution and cultured under standard cell culture conditions (37 °C with 5% CO_2_) for 8 h without shaking. Bacteria were then harvested and washed twice with ice-cold PBS, and heat inactivated at 60°C for 30 min. Complete killing of bacteria was confirmed by 72 h incubation at 37 °C on bacteria growth plates.

### *C. rodentium* infection

*C. rodentium* were grown overnight in LB broth supplemented with Km (50 μg ml^−1^) with shaking at 37 °C. Mice were infected by oral gavage with 0.2 ml of PBS containing ∼1 × 10^9^ c.f.u. (colony-forming unit) of *C. rodentium*. To determine bacterial numbers in the faeces, faecal pellets were collected from individual mice, homogenized in cold PBS, and plated at serial dilutions onto MacConkey agar plates containing 50 μg ml^−1^ Km, and the number of c.f.u. was determined after overnight incubation at 37 °C. To deplete CCR2^+^ monocyte and monocyte-derived macrophages *in vivo*, 10 ng g^−1^ body weight of diphtheria toxin was injected intraperitoneally on day 5, 7, 9 and 11 post infection.

### Flow cytometric analysis

Flow cytometry was performed using a FACSCanto II or FACSAria III and analysed using FlowJo software (TreeStar). Dead cells were excluded with 7-AAD staining. Non-specific antibody binding was blocked with anti-CD16/32 antibody. Fluorescence-conjugated mAb against CD11b (M1/70), CD11c (N418), Gr-1 (RB6-8C5), F4/80 (BM8), Ly6C (AL-21), MHC class II I-Ab (AF6-120.1), CD103 (2E7), CD45 (30-F11), CD3 (145-2C11), CD4 (GK1.5), NKp46 (29A1.4), Thy-1.2 (53-2.1) and IL-22 (1H8PWSR) were from eBioscience. Isotype-matched antibodies (eBioscience) were used for control staining. All antibodies are used in 1:200 dilution in 1 × 10^6^ cells per 100 μl except Gr-1, Ly6C, I-Ab and CD45 (used in 1:500 dilution).

### Preparation of lamina propria mononuclear cells

Lamina propria mononuclear cells (LPMCs) were isolated from intestinal specimens using modifications of previously described techniques^57^. Briefly, dissected mucosa was incubated in calcium and magnesium-free Hank's balanced salt solution (HBSS) (Sigma-Aldrich) containing 2.5% heat-inactivated fetal bovine serum (FBS) and 1 mM dithiothreitol (Sigma-Aldrich) to remove mucus. The mucosa was then incubated twice in HBSS containing 1 mM EDTA (Sigma-Aldrich) for 45 min at 37 °C. Tissues were collected and incubated in HBSS containing 400 U ml^−1^ collagenase type 3 and 0.01 mg ml^−1^ DNase I (Worthington Biochemical) for 90–180 min at 37 °C. The fraction was pelleted and resuspended in a 40% Percoll solution (Amersham Biosciences), then layered on 75% Percoll gradient before centrifugation at 2,000 r.p.m. for 20 min at room temperature. Viable LPMCs were recovered from the 40 to 75% layer interface.

### Isolation of subsets of intestinal cells

For isolation of intestinal Th17 cells and ILC3, LPMCs were isolated from RORγt^GFP/+^ reporter mice. CD11b-, CD11c-, CD19-, B220-, CD49b-, CD105-, MHC-II- and Ter-119-expressing cells were depleted from LPMCs using a pan T-cell isolation kit (Miltenyi Biotech). Enriched LP cells were then stained for CD4 and CD3. Th17 (CD3^+^CD4^+^RORγt^+^) and ILC3 (CD3^-^RORγt^+^) were sorted using a FACSAria III cell sorter. For isolation of intestinal antigen-presenting cells subsets, LPMCs were isolated from infected- and uninfected-CD115^GFP^ reporter mice. CD115^hi^Gr-1^−^CD11c^+^CD11b^+^ cells (MP1) and CD115^lo^Gr-1^−^CD11c^+^CD11b^−^ cells (DC1) were sorted using a FACSAria III cell sorter.

### Intestinal cell culture

Total LPMCs or sorted intestinal cell subsets were resuspended in RPMI medium containing 10% FBS, 1% penicillin/streptomycin, 2-mercaptoethanol (50 μM), L-glutamine (2 mM), sodium pyruvate (1 mM), HEPES (1 mM) and MEM non-essential amino acids (Gibco) at 2 × 10^6^ cells per ml (LPMC) or 1 × 10^6^ cells per ml (sorted cells). LP cells were then incubated for 24 h at 37 °C with or without LPS (100 ng ml^−1^), CpG (5 μM) or heat-killed *C. rodentium* (at a bacteria:host cell ratio of 10). Culture supernatants were harvested and cytokine levels were measured by ELISA. In some experiments, recombinant cytokines or neutralizating antibodies were added to cell cultures. For intracellular cytokine staining, LP cells were cultured for 16 h with heat-killed *C. rodentium*. Brefeldin A (BD bioscience) was added in the last 2 h of incubation and LP cells were stained, fixed and permeabilized for intracellular staining using Cytofix/Cytoperm buffer (BD Biosciences).

### Quantitative real-time PCR

RNA was extracted using E.N.Z.A. Total RNA Kit (Omega Biotek) according to manufacturer’s instructions. Purified RNA was reverse transcribed using the High Capacity RNA-to-cDNA kit (Applied Biosystem). The cDNA was then used for quantitative PCR by the SYBR Green Gene Expression Assay (Applied Biosystem) on BioRad CFX Connect Real-Time PCR System. Primer sequences were provided in Supplementary Table 1.

### BMDM *in vitro* stimulation

BM cells were cultured for 5 days with Iscove’s Modified Dulbecco’s Medium (IMDM) supplemented with 30% L929 supernatant containing macrophage-stimulating factor, glutamine, sodium pyruvate, 10% FBS and antibiotics. Differentiated BMDMs were then washed and cultured in antibiotic free IMDM supplemented with 2% FBS. BMDMs (2 × 10^5^ cells/well/48 well plate in 200 μl) were stimulated with individual bacterial strains at a multiplicity of infection (MOI)=25 for 1 h followed by 17 h of additional culture in the presence of gentamicin (100 μg ml^−1^) to prevent bacterial overgrowth. In some experiments, BMDMs were pretreated with LPS (100 ng ml^−1^) for 3 h before bacteria stimulation. Culture supernatants were harvested and cytokines were measured by ELISA.

### Monocyte adoptive transfer

BM cells were harvested, washed in FACS buffer and monocytes were isolated by the EasySep Mouse Monocyte Enrichment Kit (STEMCELL Technologies). Monocytes were counted and transferred by intravenous injection of 2 × 10^6^ in the tail vein of *Ccr2*^*−/−*^ mice 4 days after oral infection with *C. rodentium*.

### Mixed BM chimera mice

To generate mixed BM chimera mice, 10–12 week-old WT recipient mice were lethally irradiated using X-rays (Phillips RT250, Kimtron Medical) with two doses of 540 rads (total 1,080) delivered 3 h apart on the same day, then 5 × 10^6^ of BM cells from CCR2^WT^ or CCR2^DTR^ mice mixed with 5 × 10^6^ of BM cells from *Il1b*^*−/−*^ mice and transplanted into recipient mice via tail vein injection. Recipient mice were rested for at least 8 weeks before use.

### Immunoblotting of caspase-11

The CD45^+^MHC-II^+^CD11b^+^CD11c^+^CD103^-^Gr-1^−^ MP1 cell subset was purified from LPMCs isolated from naive or *C. rodentiun*-infected (day 8) mice using a FACSAria cell sorter. FACS purified cells (2 × 10^5^ cells) were lysed in a buffer containing 1% NP-40 supplemented with the complete protease inhibitor cocktail (Roche, Mannheim, Germany) and 2 mM dithiothreitol. Lysates were resolved by SDS–polyacrylamide gel electrophoresis and transferred to PVDF (polyvinylidene difluoride) membranes by electro-blotting. Rat anti-mouse-caspase-11 antibody (17D9, Sigma) was used to detect caspase-11 in MPs (1:1000 dilution).

### Statistical analyses

Statistical analyses were performed using GraphPad Prism software version 5.0 (GraphPad Software). Differences between two groups were evaluated using Student’s *t*-test (parametric) or Mann–Whitney *U*-test (non-parametric). For the comparison of more than three groups, statistical analysis was performed using one-way analysis of variance (parametric) or Kruscal–Wallis test (non-parametric), and then the Dunnett’s or Bonferroni test for parametric samples, or Dunn’s test for non-parametric samples as a post-hoc test. Survival between groups of mice was compared using Log-rank (Mantel–Cox) test. Differences at *P*<0.05 were considered significant.

## Additional information

**How to cite this article:** Seo, S.-U. *et al.* Intestinal macrophages arising from CCR2^+^ monocytes control pathogen infection by activating innate lymphoid cells. *Nat. Commun.* 6:8010 doi: 10.1038/ncomms9010 (2015).

## Supplementary Material

Supplementary Information Supplementary Figures 1-12 and Supplementary Table 1
