# Supplementary material for: Intestinal macrophages arising from CCR2+ monocytes control pathogen infection by activating innate lymphoid cells
Source: Nat Commun. 2015 Aug 13;6:8010. doi: 10.1038/ncomms9010 (PMC4536571; doi:10.1038/ncomms9010)
Supplement: Supplementary Information — Supplementary Figures 1-12 and Supplementary Table 1 [file ncomms9010-s1.pdf]

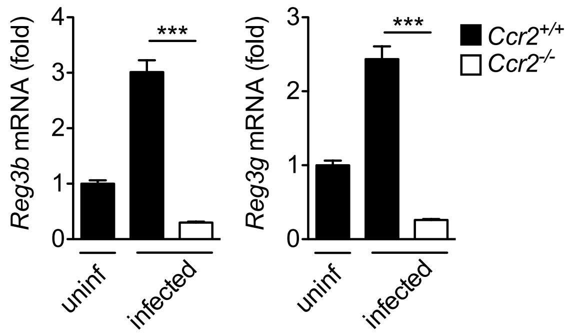

Supplementary Figure 1. RegIIIβ/γ induction is regulated by recruitment of CCR2+ monocytes.
Colonic epithelial cells were isolated from C. rodentium infected (8 dpi) and uninfected *Ccr2*$^{+/+}$ and *Ccr2*$^{-/-}$ mice. Expression of Reg3β and Reg3γ mRNA was analyzed by qPCR. Expression of target genes was normalized to Gapdh. Data are given as mean ± s.d. (n=3). ***p<0.001 by Student's t test.

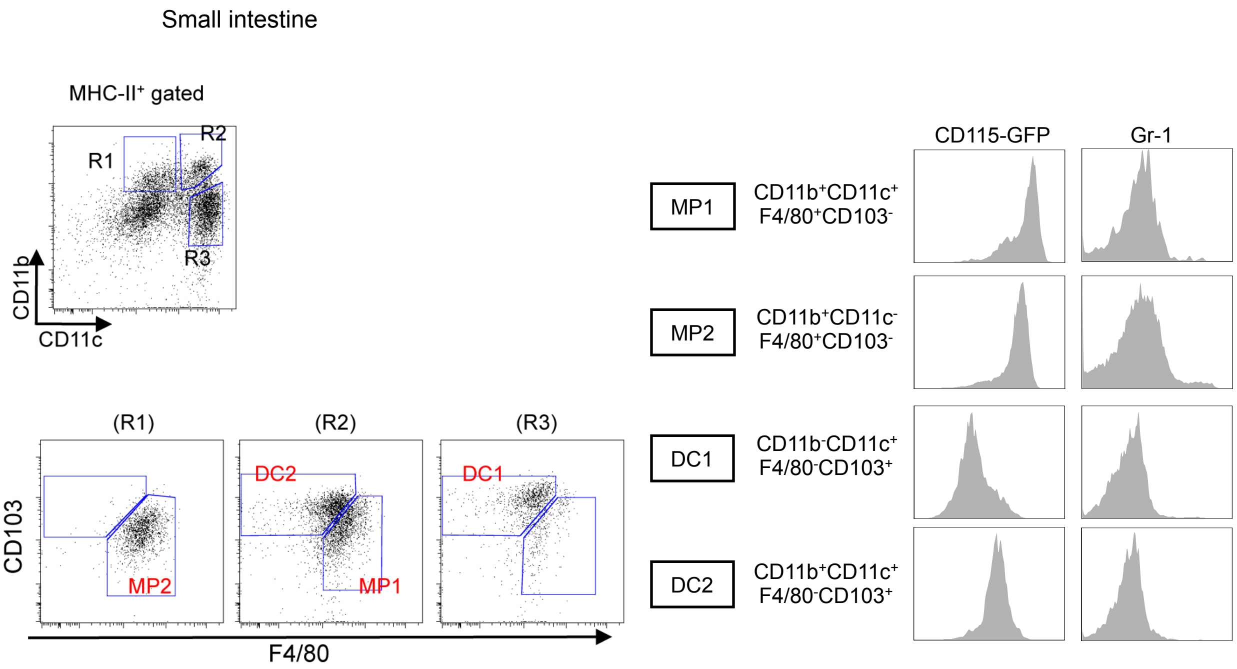

Supplementary Figure 2. Analysis of subsets of mononuclear phagocytes in the small intestine.
Total LP cells were isolated from the small intestine of uninfected CD115^{GFP} reporter mice. CD45^{+}MHC-II^{+} mononuclear phagocytes from CD115^{GFP} mice were further analyzed by flow cytometry.

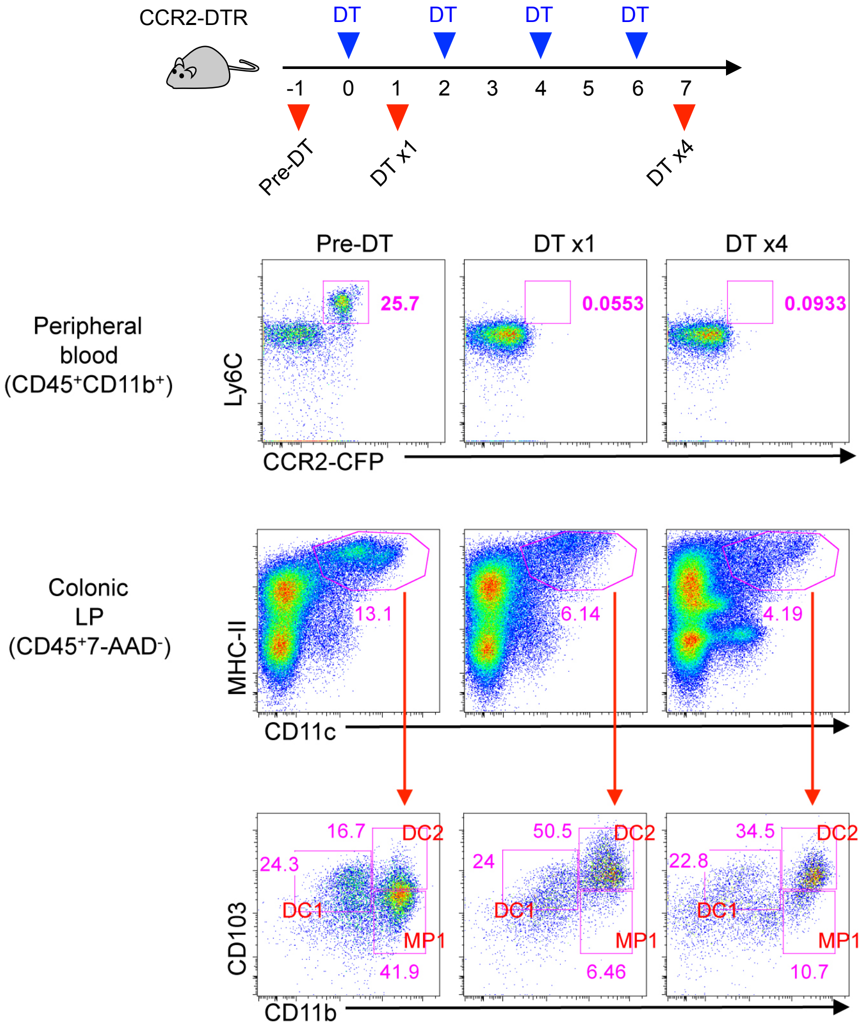

Supplementary Figure 3. Selective depletion of MP1 subset in CCR2^{DTR} mice after single and multiple injections of DT.
Uninfected CCR2^{DTR} mice were injected with diphtheria toxin (DT; 10 ng g^{-1} body weight) 4 times in 2 day intervals. Colonic LP cells were isolated pre-injection, on day 1 post injection (single injection), and on day 7 (post 4 injections), and colonic macrophage and DC subsets were analyzed. Results are representative of at least 2 individual mice.

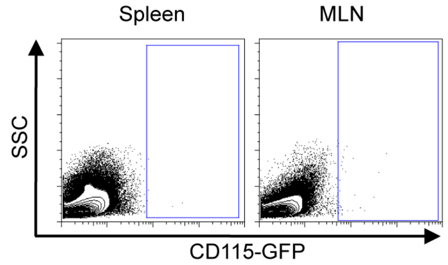

Supplementary Figure 4. Transferred monocytes do not migrate to extra-intestinal lymphoid tissues.

Ccr2$^{-/-}$ mice were infected with *C. rodentium*. On day 4 post-infection, CD11b^{+}Ly6C^{hi} bone-marrow monocytes were isolated from CD115-GFP mice and transferred into *Ccr2*$^{-/-}$ recipient mice as described in Figure 2d. The presence of GFP^{+} cells in spleen and MLN (day 10 post transfer) was assessed. Results are representative of 2 independent experiments.

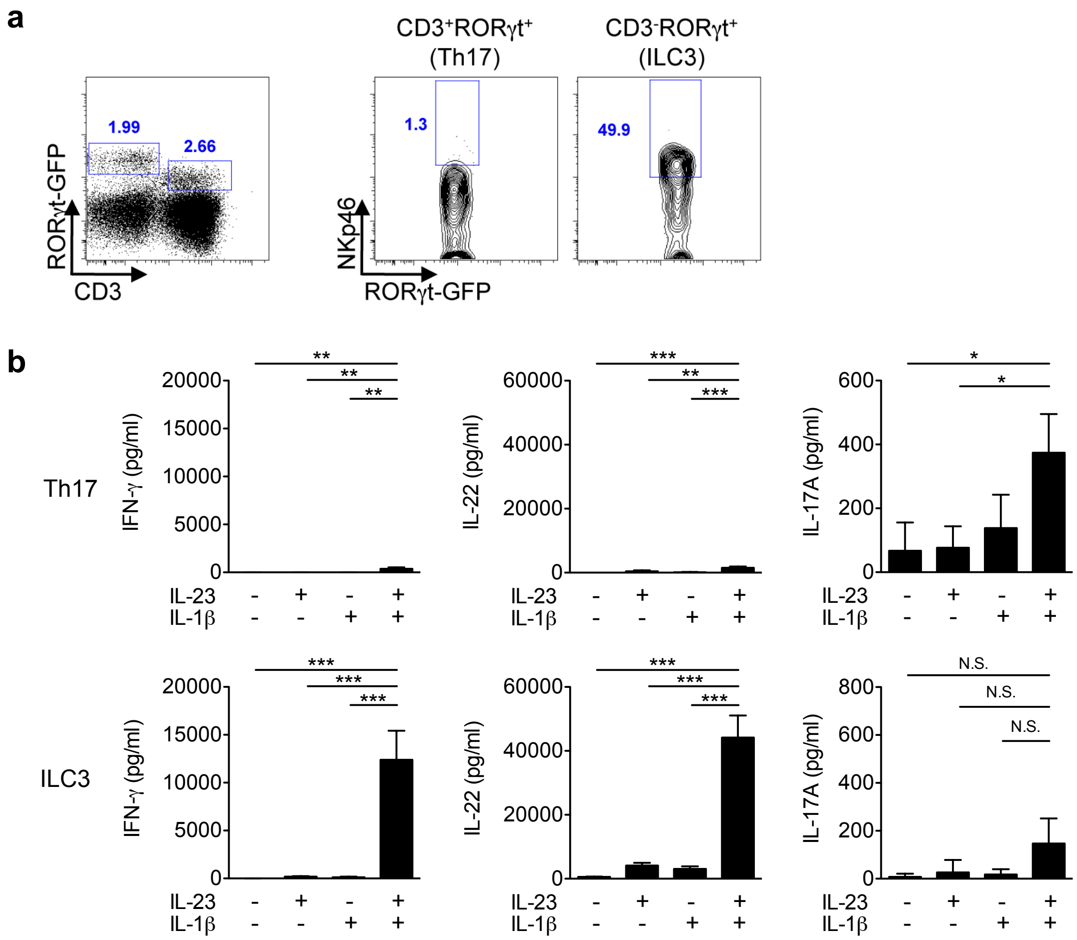

Supplementary Figure 5. RORγt+ ILC3s produce robust amounts of IL-22 and IFN-γ in response to IL-23 and IL-1β.

(**a**) LPMCs were isolated from RORγt^{GFP} reporter mice and $CD3^{+}RORgt^{+}$ (Th17) and $CD3^{-}RORγt^{+}$ (ILC) cells were further stained for NKp46. (**b**) Purified intestinal $CD3^{+}CD4^{+}RORγt^{+}$ Th17 cells and $CD3^{-}RORgt^{+}$ ILCs (1 x 10^{6} cells ml^{-1}) were stimulated with recombinant IL-23 (20 ng ml^{-1}), IL-1β (20 ng ml^{-1}), or IL-23 plus IL-1β for 24 hrs. Cytokines in culture supernatants were analyzed by ELISA. Data are given as mean ± s.d. (n=4). *p<0.05; **p<0.01;***p<0.001; N.S., not significant by Bonfferoni test.

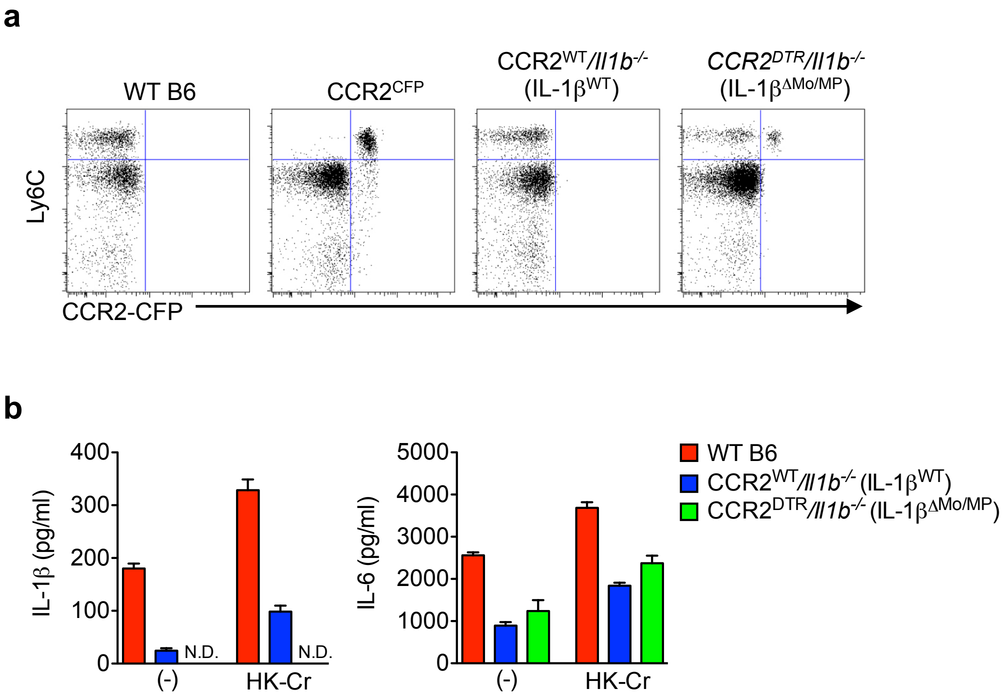

Supplementary Figure 6. CCR2^{WT}/*Il1β*$^{-/-}$ mixed chimeric mice produce detectable but impaired level of IL-1β compared to non-chimeric control mice.

(**a**) Peripheral blood were isolated from WT, CCR2^{WT}/*Il1β*$^{-/-}$ (IL-1β^{WT}) and CCR2^{DTR}/*Il1β*$^{-/-}$ (IL-1β$^{\Delta Mo/MP}$) chimeric mice. Expression of CCR2-CFP was analyzed on CD45$^+$CD11b $^+$Ly6C^{hi} monocytes. (b) WT, *Ccr2*^{WT}/*Il1β*$^{-/-}$ (IL-1β^{WT}) and *Ccr2*^{DTR}/*Il1β*$^{-/-}$ (IL-1β$^{\Delta Mo/MP}$) mice were infected with *C. rodentium*, and CCR2$^+$ monocytes and monocyte-derived MP1 cells were depleted by DT injection (10 ng/g body weight) on days 5 and 7 post-infection. On day 8 post infection, colonic LPMCs were isolated. 2 x 10^6 cells ml^{-1} LPMCs were cultured in the presence or absence of heat-killed *C. rodentium* (MOI=10) for 24 hrs. Cytokines in culture supernatants were analyzed by ELISA. Data are given as mean ± s.d. (n=3).

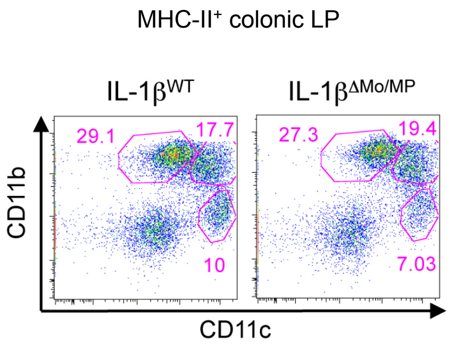

Supplementary Figure 7. Analysis of CCR2^{DTR}/*Il1b*$^{-/-}$ mixed chimeric mice colonic macrophages.

CCR2^{WT}/*Il1b*$^{-/-}$ (IL-1β^{WT}) or CCR2^{DTR}/*Il1β*$^{-/-}$ (IL-1β$^{\Delta Mo/MP}$) mice were infected with *C. rodentium*, and CCR2^{+} monocytes and monocyte-derived MP1 cells were depleted by DT injection (10 ng g^{-1} body weight) on days 5 and 7 post-infection. On day 8 post infection, colonic LPMCs were isolated, and colonic macrophage and DC subsets were analyzed. The percentage of colonic macrophages and DCs were comparable between IL-1β^{WT} and IL-1β$^{\Delta Mo/MP}$ mice.

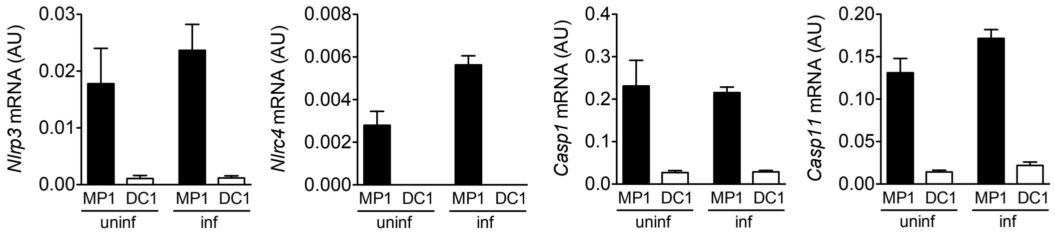

Supplementary Figure 8. Inflammasome activation in MP1 cells.
MP1 and DC1 subsets were isolated from uninfected and *C. rodentium*-infected CD115-GFP animals. Cytokine mRNA expression was analyzed by qPCR. Data are given as mean ± s.d. (n=5-7).

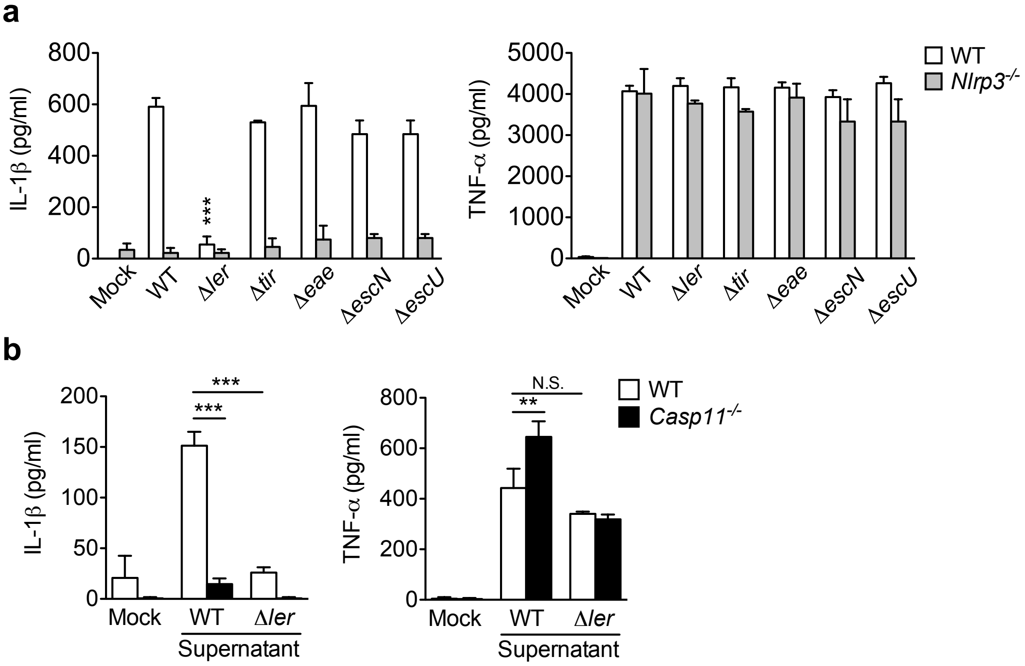

Supplementary Figure 9. Ler-dependent virulence factors in *C. rodentium* is required for caspase-11-mediated IL-1β induction.
(**a**) BMDMs from WT and *Nlrp3*$^{-/-}$ mice were stimulated with WT *C. rodentium* or isogenic mutants (MOI=25) 1 hr without antibiotics and then cultured additional 17 hrs in the presence of 100 μg ml^{-1} gentamicin. Cytokines in the culture supernatant were analyzed by ELISA. Data are given as mean ± s.d. (n=3). Results are representative of 3 independent experiments. *** $p<0.001$ by Dunnett's test (compared to WT). (**b**) WT and Δ*ler* mutant *C. rodentium* were cultured in DMEM in cell culture incubator for 16 hrs. Bacterial culture supernatants were harvested and live bacteria were removed by passing 0.45 μm syringe filter. One third volume of sterile bacterial culture sups were added in BMDMs from WT and *Casp11*$^{-/-}$ mice, and incubated for 18 hrs. Cytokines in the culture supernatant were analyzed by ELISA. mean ± s.d. (n=3). Results are representative of 3 independent experiments. **$p<0.01$; ***$p<0.001$; N.S., not significant by Bonfferoni test.

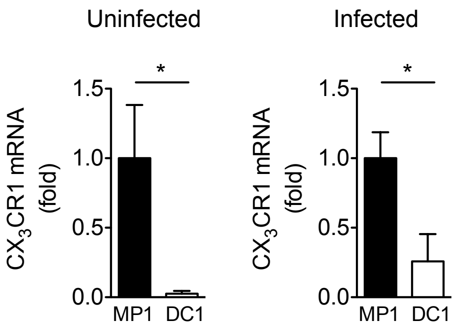

Supplementary Figure 10. CX_3CR1 expression in MP1 and $CD103^+$ DC subsets.

MP1 and DC1 subsets were isolated from uninfected and *C. rodentium*-infected $CD115^{gfp}$ animals. Expression of CX_3CR1 was analyzed by qPCR. Data are given as mean ± s.d. (n=5-7). *$p<0.05$; by Mann-Whitney *U* test.

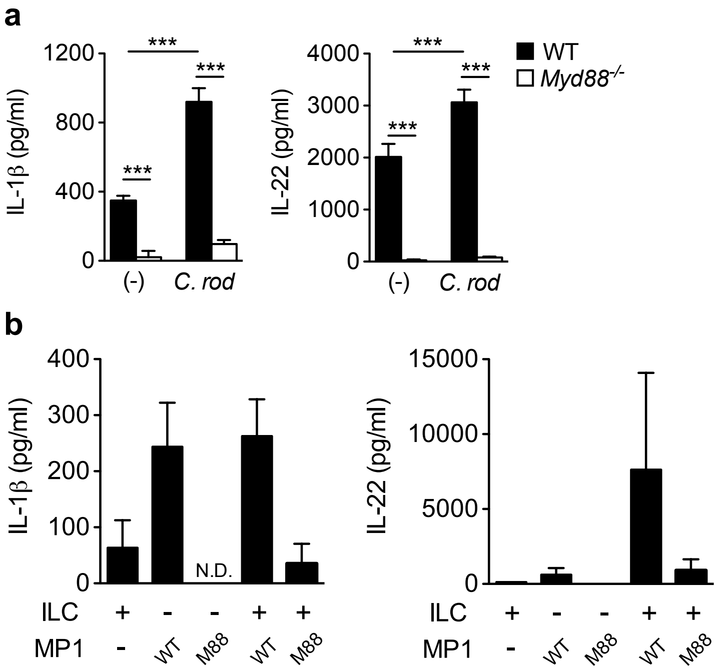

Supplementary Figure 11. MyD88 signaling is required for IL-1β production and ILC activation by MP1.

(**a**) WT and *Myd88*$^{-/-}$ mice were infected with *C. rodentium*. On day 8 post infection, colonic LPMCs were isolated. 2 x 10^6 cells ml^{-1} LPMCs were cultured in the presence or absence of heat-killed *C. rodentium* (MOI=10) for 24 hrs. Cytokines in culture supernatants were analyzed by ELISA. Data are given as mean ± s.d. (n=3). Results are representative of 3 independent experiments. *** $p<0.001$ by Dunn's test. (**b**) $CD3^-$ $ROR\gamma t^+$ ILCs from uninfected $ROR\gamma t^{GFP/+}$ reporter mice and MP1 cells from *C. rodentium*-infected (day 8) WT and *Myd88*$^{-/-}$ mice were isolated. ILCs and MP1 cells (1 x 10^6 cells ml^{-1}) were cultured alone or co-cultured with heat-killed *C. rodentium* (MOI=10) for 24 hrs. Data are given as mean ± s.d. of 3 independent experiment.

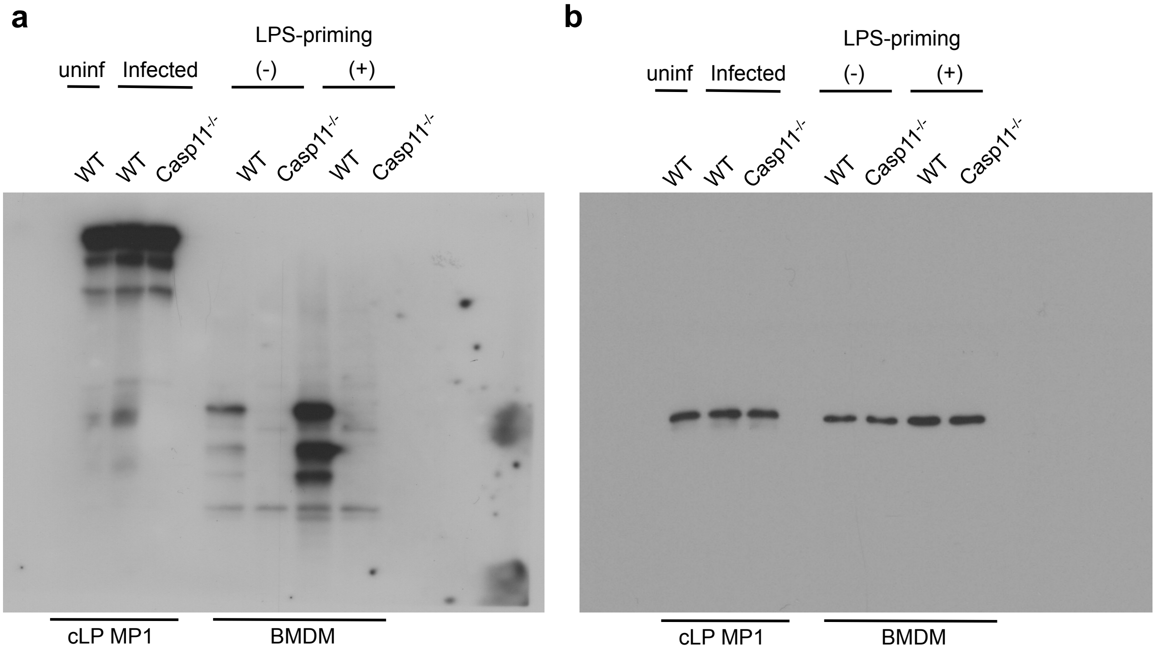

Supplementary Figure 12. The original images for the main figures.
(**a**) Fig. 5e; Caspase-11. (**b**) Fig. 5e; β-actin

Supplementary Table 1. Primer sequences used in this study.

Genes	Forward	Reverse
IL-1β	5'-CAACCAACAAGTGATATTCTCCATG-3'	5'-GATCCACACTCTCCAGCTGCA-3'
IL-23p19	5'-TCCCTACTAGGACTCAGCCAAC-3'	5'-GCTGCCACTGCTGACTAGAA-3'
IL-12p35	5'-CCAGGTGTCTTAGCCAGTCC-3'	5'-GCAGTGCAGGAATAATGTTTCA-3'
IL-12/23p40	5'-CCTGAAGTGTGAAGCACCAAATTAC-3'	5'-GAACTTCAAGTCCATGTTTCTTTGC-3'
IL-6	5'-GAGGATACCACTCCCAACAGACC-3'	5'-AAGTGCATCATCGTTGTTCATACA-3'
TNF	5'-GCCTCCCTCTCATCAGTTCT-3'	5'-CACTTGGTGGTTTGCTACGA-3'
IL-10	5'-CCCTTTGCTATGGTGTCCTT-3'	5'-TGGTTTCTCTTCCCAAGACC-3'
TGF-b1	5'-TGACGTCACTGGAGTTGTACGG-3'	5'-GGTTCATGTCATGGATGGTGC-3'
IL-22	5'-TTTCCTGACCAAACTCAGCA-3'	5'-TCTGGATGTTCTGGTCGTCA-3'
NLRP3	5'-ATGGTATGCCAGGAGGACAG-3'	5'-ATGCTCCTTGACCAGTTGGA-3'
NLRC4	5'-AGAAGGGCTCAGCGGCCTGCAA-3'	5'-TTCACCCAGGGGGTAGAAGTTCA-3'
Caspase-1	5'-GCCCACTGCTGATAGGGTGA-3'	5'-CCCGGGAAGAGGTAGAAACG-3'
Caspase-11	5'-TGTCATCTCTTTGATATATTCCTGAAG-3'	5'-CAAGGTTGCCCGATCAAT-3'
RegIIIβ	5'-CTCTCCTGCCTGATGCTCTT-3'	5'- GTAGGAGCCATAAGCCTGGG-3'
RegIIIγ	5'- TCAGGTGCAAGGTGAAGTTG-3'	5'-GGCCACTGTTACCACTGCTT-3',
CX_3CR1	5'-AAGTTCCCTTCCCATCTGCT-3'	5'-CAAAATTCTCTAGATCCAGTTCAGG-3'
β-actin	5'-AAGTGTGACGTTGACATCCG-3'	5'-GATCCACATCTGCTGGAAGG-3'
